# Bioenergetics in human evolution and disease: implications for the origins of biological complexity and the missing genetic variation of common diseases

**DOI:** 10.1098/rstb.2012.0267

**Published:** 2013-07-19

**Authors:** Douglas C. Wallace

**Affiliations:** Center of Mitochondrial and Epigenomic Medicine, Children's Hospital of Philadelphia, Department of Pathology and Laboratory Medicine, University of Pennsylvania, Colket Translational Research Building, Room 6060, 3501 Civic Center Boulevard, Philadelphia, PA 19104-4302, USA

**Keywords:** mitochondria, bioenergetics, evolution

## Abstract

Two major inconsistencies exist in the current neo-Darwinian evolutionary theory that random chromosomal mutations acted on by natural selection generate new species. First, natural selection does not require the evolution of ever increasing complexity, yet this is the hallmark of biology. Second, human chromosomal DNA sequence variation is predominantly either neutral or deleterious and is insufficient to provide the variation required for speciation or for predilection to common diseases. Complexity is explained by the continuous flow of energy through the biosphere that drives the accumulation of nucleic acids and information. Information then encodes complex forms. In animals, energy flow is primarily mediated by mitochondria whose maternally inherited mitochondrial DNA (mtDNA) codes for key genes for energy metabolism. In mammals, the mtDNA has a very high mutation rate, but the deleterious mutations are removed by an ovarian selection system. Hence, new mutations that subtly alter energy metabolism are continuously introduced into the species, permitting adaptation to regional differences in energy environments. Therefore, the most phenotypically significant gene variants arise in the mtDNA, are regional, and permit animals to occupy peripheral energy environments where rarer nuclear DNA (nDNA) variants can accumulate, leading to speciation. The neutralist–selectionist debate is then a consequence of mammals having two different evolutionary strategies: a fast mtDNA strategy for intra-specific radiation and a slow nDNA strategy for speciation. Furthermore, the missing genetic variation for common human diseases is primarily mtDNA variation plus regional nDNA variants, both of which have been missed by large, inter-population association studies.

## 1. Evolution and energetics

In the mid-nineteenth century, Charles Darwin and Alfred Russel Wallace proposed that species arose through natural selection, thus accounting for how one species can split into two similar species occupying adjacent niches. Darwin & Wallace [1,2] elaborated on this theory to explain many important biological questions. However, Wallace was concerned that natural selection was insufficient to explain the origins of consciousness and the human brain. At the time and even today, the primary debate is whether biology is a science that can be understood by natural principles. Hence, Wallace's concerns have been minimized by the natural scientists.

With the maturation of physical science theories–in particular, thermodynamics–an inconsistency arose. Thermodynamics taught that, in a closed system, entropy increases and complexity decays. Therefore, the stable existence of complex living organisms seemed to defy physics. This dilemma was partially resolved by Schrödinger, who pointed out that living organisms were not closed systems, but rather acquire energy from their environment as what he called ‘negative entropy’ [3]. The concept that the flow of energy through a system can generate and sustain complex structures is the domain of non-equilibrium thermodynamics that is now understood to be central to life, the reason why we eat and breathe [4–6].

Still, energy flow only explains the maintenance of complexity. It does not necessitate the development of complex forms. Resolution of this dilemma comes from information theory. The discovery of the structure of DNA revealed that biology is primarily about the storage and retrieval of information. Within a narrow thermal range, such as exists on Earth, the flow of energy through organic systems generates ordered structures. One of these structures is nucleic acids, which can accumulate information. Each year, the flow of energy through the biosphere from sunlight on the Earth's surface or from geothermal vents on the ocean floor provides the energy to generate more nucleic acids, and the more nucleic acids the more information, the more information the greater the complexity [7]. Originally, energy flow created nucleic acids directly, though inefficiently [6,8]. With the advent of intra-cellular DNA replication, however, nucleic acids accumulated rapidly through cell proliferation. Hence, the complexity of modern organisms is a consequence of four billion years of energy flow and the resulting information accumulation, making biological information stored energy. This implies that one of the more important actions of natural selection is enrichment for the more energy-efficient individuals among organisms attempting to exploit the same energy resource [7].

## 2. Evolution and Mendelian genetics

In the later part of the nineteenth century, Gregor Mendel outlined the rules of inheritance for sexually reproducing organisms. Each parent provides one copy of each gene to an offspring through fusion of the male and female gametes, generating a ‘diploid’ individual. At sexual maturity, the individual separates the two gene copies into his/her sex cells in preparation for conception of the next generation.

Subsequently, it was discovered that the behaviour of the genes corresponded to the behaviour of the chromosomes, and later that chromosomes packaged DNA. Since nucleic acids can replicate and in the process mutate, this led to the concept that mutations in the nDNA generate the variation that is acted on by natural selection to create organismal diversity. This neo-Darwinian synthesis implied that a significant proportion of the nDNA variation must be functional and, in the right environment, beneficial.

This concept stood until the 1960s, when molecular genetic studies on human nDNA revealed that much of the genetic variation that differentiated human populations is due to differences in the frequency of alleles common to both populations. Furthermore, the differences in allelic frequencies between populations could be explained primarily by statistical fluctuations [9–11]. This led Kimura to propose that virtually all extant chromosomal genetic variation was neutral, because the vast majority of functional mutations would be deleterious and removed by purifying selection [12]. This ‘neutralist’ hypothesis precipitated the neutralist–selectionist debate. If all intra-specific genetic variation was neutral, where was the functional variation that could permit individuals to adapt to environmental changes and ultimately give rise to new species?

Since before the time of Darwin and Wallace, species have been defined primarily by anatomical differences and all anatomical traits are coded by nDNA genes. Consequently, analysis of nDNA variation has been highly informative in understanding the progressive changes in anatomical traits that occur during speciation. By contrast, so much of human nDNA variation could be explained by stochastic processes that it soon became dogma that all intra-specific genetic differences were the result of stochastic processes such as genetic drift and founder effects.

However, humans are a single species and anatomy does not vary markedly within a species. Therefore, intra-specific variation must affect functions other than anatomical traits. Since energy is also fundamental to the life process, bioenergetic changes could be the source of intra-specific variation.

A new opportunity arose to find adaptive variation within the human nDNA as a consequence of the Human Genome Project. The screening of multiple human nDNAs permitted the identification of millions of single nucleotide polymorphisms (SNPs). These SNPs were then used to screen populations to identify chromosomal regions that are linked (in linkage disequilibrium) with loci that alter the risk of developing metabolic diseases. Major human metabolic phenotypes include diabetes and obesity and these clinical manifestations are directly related to individual responses to environmental differences in energy resources. In fact, many important environmental differences are related to the type and availability of calories and demands for use of those calories for tissue maintenance, physical work, combating infections, reproduction and to cope with environmental limitations such as oxygen deprivation and toxins. The aggregate of all of these factors will be referred to here as the energetic environment. Since the energetic environment within a species' niche can change, chromosomal locus variants that are beneficial at one time in one energetic environment might become deleterious in another. Therefore, chromosomal loci related to diabetes and obesity should provide insight into the genetics of energy metabolism.

Genome Wide Association Studies (GWAS) have identified 63 chromosomal loci associated with type 2 diabetes. However, all of these loci have very weak phenotypic effects, so in aggregate they account for only 5.7 per cent of the variance in disease susceptibility. Simulation studies have suggested that an additional 488 loci may contribute to type 2 diabetes risk, but again the aggregated effect of all such loci would still explain only 10.7 per cent of risk. Additional projections suggest that if it were technically possible to identify them, perhaps approximately 49 per cent of the risk variance might be explained by common variants with low phenotype effect [13]. Taking into account body mass index, additional insulin resistance loci have been identified [14], but the chromosomal variants found by GWAS still fall far short of accounting for the approximately 3.5-fold increased risk faced by first degree relatives of diabetes patients [15].

Since the GWAS study design relies on linked DNA polymorphisms to identify disease risk loci, it requires that one or more SNPs be stably linked to the functional locus. Because the observed loci have weak phenotypic effects, large numbers of samples have been required to achieve statistical significance. To obtain sufficient numbers, successful studies have combined the data from multiple geographically dispersed populations. This research design requires that the DNA polymorphism must have become associated with the functional locus early in human radiation so that the resulting linkage unit could become dispersed throughout global populations. Given that severely deleterious mutants would be eliminated by purifying selection, this research design necessitates that only the variants with the most modest phenotypic effects would survive long enough to remain associated within a single linkage group and become sufficiently dispersed throughout human populations to be detected by GWAS studies.

Since loci with large phenotypic effects would be rapidly eliminated by purifying selection, any extant high effect locus must be of recent origin. Having arisen recently, such large phenotypic effect loci must be confined to a single regional population and linked to a set of SNPs that are not associated with this functional locus anywhere else in the world. As a result, analysis of the relevant SNPs in a large multiple population study would dilute out any association between chromosomal SNPs and important regional phenotypic variants.

Such large phenotypic effect loci for diabetes and obesity loci have often been reported in regional association studies. One group of notable gene variants are those associated in the uncoupling protein (UCP) 1–3 genes [16–19]. However, these population-specific associations have often been dismissed as being ‘non-replicateable’ in other populations. But population-specific associations are precisely what would be expected for the most phenotypically significant gene variants.

One important type 2 diabetes locus is the peroxisome-proliferating-activated receptor γ (PPARγ) gene that has been associated with diabetes in GWAS studies [13,20,21]. However, a specific PPARγ variant, P121A, has also been associated with diabetes in specific populations and validated through family studies [22]. Hence, this locus encompasses both ancient, small-effect variants as well as recent, regional, large-effect variants. A G482S amino acid substitution in the functionally related PPAR*γ* coactivator gene-1α (PGC-1α) gene has also been associated with metabolic alterations in regional populations, diabetes in the Danish [23] and altered lipid metabolism in the Pima Indians [24], but PGC-1α is not routinely detected by GWAS [13].

These observations suggest that there is a continuum of phenotypic effects among nDNA bioenergetic gene variants. The milder mutant phenotypes, which are less affected by purifying selection, may be retained for prolonged periods in the human population. Those that arose early in human radiation have remained with their original linkage group while being dispersed throughout the global population, and provide a signal in inter-population GWAS studies. More phenotypically significant variants have arisen throughout human history but have been acted on by purifying selection, eliminating them from the global population. Hence, these loci are not associated with widely dispersed chromosomal linkage groups. Some mutants, such as the PGC-1α G482S mutation, may be sufficiently adaptive to have arisen multiple independent times in different populations on different haplotypes, each new variant associated with a different linkage group rendering them undetectable by GWAS. To find these regional high impact variants it will be necessary to study regional populations for functional genetic mutations, presumably by whole-genome sequencing.

## 3. Evolution and energetics

PPARγ and PGC-1α are nuclear transcription factors that play a major role in regulating bioenergetics and particularly mitochondrial biogenesis. The UCPs regulate the coupling efficiency of the mitochondrial energy production system, oxidative phosphorylation (OXPHOS). Hence, the importance of these and multiple other loci identified by GWAS implicate mitochondrial bioenergetics in diabetes and obesity and, by extension, mitochondrial functional variation in human regional environmental adaptation. Consistent with this supposition, the mitochondria are estimated to generate about 90 per cent of the cellular energy in differentiated tissue cells and the mitochondrial genome encompasses in the order of one to two thousand nDNA genes and thousands of copies of the mtDNA. Hence, a large number of mitochondrial gene targets can be mutated and have significant effects on cellular bioenergetics.

The mitochondria are the product of a symbiosis between two micro-organisms that occurred about two billion years ago. The nature of the original partner organisms is actively debated, but the progenitor of the mitochondrion is thought to have been an α-protobacterium that harboured a complete OXPHOS system. Both of these organisms alone were limited in their complexity since a single bacterial cell can generate only enough energy to sustain about 10 000 genes [5,6]. However, when the host cell acquired multiple oxidative bacteria, the bacterial energy could be pooled to provide the required energy for adding more genes to the host cell's DNA to create more complex anatomical structures. There was a problem, however. The oxidative bacteria needed most of their energy to sustain themselves. This dilemma was resolved since bacteria readily exchange genes. By transferring structural genes from the oxidative bacteria to the host cell's DNA, the number of bacterial gene copies was reduced from thousands to two, the pair of homologues in the nDNA. This reduced the amount of DNA to be replicated and reduced the complexity of mtDNA transcriptional regulation, with significant savings in energy. The accumulation of nDNA also permitted the nDNA genes to radiate and address new functional genetic space [5,25]. Hence, there was a significant selective pressure to transfer most of the oxidative bacterial genes to the host cell's DNA, which ultimately became the eukaryotic cell nDNA. As the number of its genes declined, the oxidative bacterium became progressively more integrated into the host cell.

Surprisingly, the progressive transfer of genes from the mtDNA to the nDNA did not go to completion and all oxidative eukaryotic cells still retain a mtDNA. In humans, the mtDNA codes for 13 polypeptide genes plus the rRNA and tRNA genes for the mitochondrial, bacteria-like, protein synthesis system [26,27].

To sustain a complete bacterial biogenesis apparatus is very energetically expensive. Therefore, there must be a strong evolutionary advantage for retaining the mtDNA. While several hypotheses have been put forward for why the mtDNA has been retained [5,28], the fact that all of the polypeptide genes retained by the mtDNA are central to the mitochondrial energy-generating system OXPHOS provides one explanation [29].

All fungal and animal mtDNAs retain essentially the same set of OXPHOS polypeptide genes. In mammals, these include seven (ND1-3, 4L, 4-6) of the approximately 45 polypeptides of the electron transport chain (ETC) enzyme complex I, one (cytochrome b, cytb) of the 11 polypeptides of the ETC complex III, three (COI-III) of the 13 polypeptides of ETC complex IV (cytochrome c oxidase, COX) and two (ATP6 and 8) of the approximately 15 polypeptides of complex V, the ATP synthase.

Functionally, the mtDNA polypeptides are central to the electron and proton wiring system for mitochondrial energy production. In OXPHOS, reducing equivalents (electrons from reduced sources) derived from food flow from reduced to oxidized down the ETC that is embedded in the mitochondrial inner membrane. Starting with NADH, which is oxidized by complex I, and succinate by complex II, the electrons are transferred to coenzyme Q (CoQ), then to complex III, then cytocrome c, then to complex IV, and finally to oxygen to generate water. As the electrons traverse complexes I, III and IV, the energy released is used to pump protons from the mitochondrial matrix across the inner membrane to the inter-membrane space. This creates an electrochemical gradient that is acid and positive on the outside and alkaline and negative on the inside [30]. The resulting capacitance of about 0.2 V is the potential energy from which virtually all human biological processes are driven. Given that a human has in the order of 10^17^ mitochondrial capacitors, this is a great deal of potential energy, the vital force that animates our life. When breathing stops, the membrane potential collapses, energy transduction ceases and death ensues [27,31,32].

The potential energy stored in the mitochondrial capacitors can be used for many purposes: to take up Ca^++^ from the cytosol, modulate cellular REDOX status and reactive oxygen species (ROS) production, and transport proteins and substrates in and out of the mitochondrion. Particularly important, however, is that the mitochondrial inner membrane potential provides the motive force for driving complex V, the ATP synthase, to condense ADP and phosphate (Pi) to generate ATP [30]. ATP is the chemical energy carrier that is exported from the mitochondrion to the cytosol to energize cellular reactions and drive work.

Given the critical nature of the inner membrane potential, it is surprising that the efficiency by which the reducing equivalents from food calories are converted into ATP differs between different individuals and regional human populations. Some individuals are highly efficient at converting caloric energy into a proton gradient via the electron transport and by transforming the energy of the proton gradient into ATP. Consequently, these individuals need to burn the least number of calories for the required ATP. Since calories are a unit of heat, such ‘tightly coupled’ individuals generate the minimum heat for the ATP used. By contrast, some individuals are less efficient at converting reducing equivalents to membrane potential, and membrane potential to ATP. These ‘loosely coupled’ individuals burn more calories for the same amount of ATP and thus produce more core body heat per ATP used. This altered coupling efficiency can be modulated epigenetically, for example, by induction of uncoupling protein 1 (UCP1) in brown fat [33], or more stably by altering the sequence of the mtDNA and thus changing proton pumping efficiency [34,35].

In tropical and temperate environments, it is generally more advantageous to be more tightly coupled so as to produce the maximum ATP with the minimum heat. However, in the arctic the constraining factor is cold. Therefore, it can be beneficial to be less coupled so that more heat is generated to maintain core body temperature, provided sufficient dietary calories are available. That this type of adaptive variation is due to mtDNA changes has been supported by the demonstration that climate differences correlate with mtDNA rather than nDNA variation [36] and that the basal metabolic rate of Siberian populations is higher than that of more southern populations [37–39].

The importance of mtDNA variation in regional adaptation makes sense when it is realized that the mtDNA codes for the proteins that are central to the coupling of electron flow to proton pumping and thus ATP production. All four mitochondrial inner membrane complexes that incorporate mtDNA-coded polypeptides (I, III, IV and V) either generate or use the proton gradient. By contrast, complex II (succinate dehydrogenase), which transports electrons but does not pump protons, is composed of four nDNA polypeptides. Since the electrochemical gradient is a capacitor, the proton permeability of complexes I, III, IV and V must be balanced with each other. If any one of the complexes becomes leaky for protons, then the capacitor can short, which would be deleterious. Hence, the 13 polypeptides of the mtDNA represent an integrated electrical circuit in which each polypeptide must be functionally compatible with the other 12 mtDNA polypeptides of comparable coupling efficiency [27].

## 4. Evolution and mtDNA genetics

Given that the mtDNA polypeptides are highly variable in their energetic efficiency [40], then the random recombination of the 13 mtDNA polypeptides between different mtDNA lineages, for example, between mtDNAs that are more or less coupled, could erode energy efficiency. This dilemma is avoided by having these variable OXPHOS electron and proton transport genes linked together in a single non-recombining piece of DNA: the maternally inherited mtDNA. This forces all of the membrane potential elements to coevolve consistent with environmental factors such as climate and diet. Uniparental inheritance of the mtDNA is achieved by the concerted elimination of the paternal mtDNA at fertilization, thus blocking inter-individual mtDNA pairing and recombination [27]. Proof that absolute uniparental inheritance of the mtDNA is critical for animals has been obtained by artificially mixing two normal but different mtDNAs within the mouse female germline. The resulting ‘heteroplasmic’ mice manifested marked behavioural abnormalities and severe learning defects [41].

**Figure 1.**
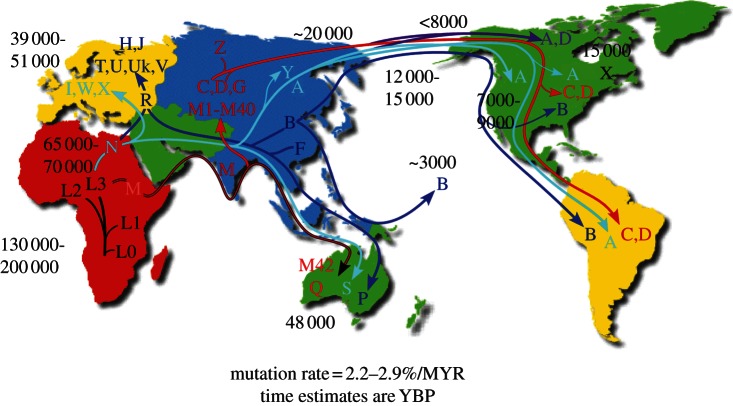
Diagram of the migratory history of the human mtDNA haplogroups. *Homo sapiens* mtDNAs arose in Africa about 130 000–200 000 years before the present (YBP), with the first African-specific haplogroup branch being L0, followed by the appearance in Africa of lineages L1, L2 and L3. In northeastern Africa, L3 gave rise to two new lineages M and N. Only M and N mtDNAs successfully left Africa about 65 000 YBP and colonized all of Eurasia and the Americas. The diverse array of mtDNA lineages that M and N spawned are clustered together as macrohaplogroups M and N. The founders of macrohaplogroup M moved out of Africa through India and along the Southeast Asian coast down along the Malaysian peninsula and into Australia, generating haplogroups Q and M42 around 48 000 YBP. Subsequently, M moved north out of Southeast Asia to produce a diverse array of mtDNA lineages including haplogroups C, D, G and many other M haplogroup lineages. In northeast Asia, haplogroup C gave rise to haplogroup Z. The founders of macrohaplogroup N also move though Southeast Asia and into Australia, generating haplogroup S. In Asia, macrohaplogroup N mtDNAs also moved north to generate central Asian haplogroup A and Siberian haplogroup Y. In western Eurasia, macrohaplogroup N founders also moved north to spawn European haplogroups I, W, and X and in western Eurasia gave rise to sub-macrohaplogroup R. R moved west to produce the European haplogroups H, J, Uk, T, U, and V and also moved east to generate Australian haplogroup P and eastern Asian haplogroups F and B. By 20 000 YBP, mtDNA haplogroups C and D from M and A from N were enriched in northeastern Siberia and thus were positioned to migrate across the Bering land bridge (Beringia) to give rise to the first Native American populations, the Paleo-Indians. Haplogroups A, C, and D migrated throughout North America and on through Central American to radiate into South America. Haplogroup X, which is most prevalent in Europe but is also found in Mongolia, though not in Siberia, arrived in North America about 15 000 YBP, but remained in northern North America. Haplogroup B, which is not found in Siberia but is prevalent along the coast of Asia, arrived in North America about 12 000 to 15 000 YBP and moved through North and Central America and into South America, combining with A, C, D and X to generate the five dominant Paleo-Indian haplogroups (A − D + X). A subsequent migration of haplogroup A out of the Chukotka peninsula about 7000 to 9000 YBP gave rise to the Na-Déné (Athabaskins, Navajo, Apache, etc.). Subsequent movement across the Bering Strait, primarily carrying haplogroups A and D after 6000 YBP, produced the Eskimo and Aleut populations. Most recently, eastern Asian haplogroup B migrated south along the Asian coast through Micronesia and out into the Pacific to colonize all of the Pacific islands. Ages of migrations are approximated using mtDNA sequence evolution rates determined by comparing regional archeological or physical anthropological data with corresponding mtDNA sequence diversity. Since selection may have limited the accumulation of diversity in certain contexts, ages for regional migrations can best be estimated from the diversity encompassed within an individual regional or continental lineage, since selection would have had its greatest effect in enriching for the founding mtDNA haplotype, after which mtDNA mutations would accumulate randomly and thus become clock-like [42,43] (reproduced from http://www.mitomap.org, with permission).

Because of strict maternal inheritance, the only way that the mtDNA sequence can change is by the sequential accumulation of mutations along radiating maternal lineages. Therefore, to a first approximation, the number of nucleotide differences between any two individuals is proportional to the time that they shared a common maternal ancestor. This unique feature of the mtDNA has permitted reconstruction of the ancient origins and migrations of women. By overlaying the mtDNA mutational tree, which shows the genetic affinities between indigenous peoples with the geographic location of the populations that harbour those mtDNAs, the progressive movement of humans was mapped (figure 1). The mtDNA proved to be a particularly powerful tool for studying human radiation, since the mtDNA sequence evolution rate was found to be much greater than that of nDNA-coded mitochondrial genes [44–46]. Indeed, the sequence evolution rate of the mtDNA has resulted in critical mtDNA changes corresponding remarkably well with the times of prominent transitional events in human geographical radiation.

The striking correlation between mtDNA variation and geographic locale of indigenous populations stands in stark contrast to nDNA variation, where the common allelic variants are dispersed across most populations, differing primarily in allele frequencies [9–11]. For the mtDNA, each indigenous population has a limited number of clusters of related mtDNA haplotypes. Each of these haplotype clusters is generally descended from a founder mtDNA that harboured one or more functional variants. Therefore, the founder mtDNAs must have arisen in the population and, as the population grew, the descendants of the founding mtDNA acquired additional variants creating population-specific groups of related mtDNA haplotypes, designated haplogroups.

Haplogroups are also related to each other in higher-order clusters [43]. All of the haplogroups in Africa, designated ‘L’ haplogroups, are components of the wider African mtDNA lineage designated macrohaplogroup L that arose between 130 000 to 200 000 years before the present (YBP) (figure 1). Of the L haplogroups, haplogroup L3 spawned two new mtDNAs, designated M and N, in the Ethiopian area. About 65 000 YBP, only mtDNAs derived from M and N successfully departed Africa and colonized the rest of the world, generating macrohaplogroups M and N. Macrohaplogroup M moved along tropical Southeast Asia, ultimately reaching Australia, and later moved north out of Southeast Asia to form a plethora of central and eastern Asian mtDNA haplogroups, including C, D, G and M1–M20. By contrast, macrohaplogroup N went in two directions. In one, N moved along Southeast Asia to Australia and from southern Asia north into central Asia to generate haplogroups A and Z. In the second, N moved due north out of Africa to form the European haplogroups I, X and W and also into western Eurasia to form sub-macrohaplogroup R. R then gave rise to the European-specific haplogroups H, J, Uk, T, U and V, and R also moved east to produce the Asian mtDNA haplogroups B and F (figure 1).

Of all of the Asian mtDNA variants, only A, C and D became enriched in northeastern Siberia and were in a position to cross the Bering Land Bridge about 20 000 YBP to give rise to the Paleo-Indians. Later, A, C and D were joined by additional migrations bringing B and X to round out the Paleo-Indian mtDNA lineages. Subsequently, additional migrations brought haplogroups A and A + D to produce the Na-Dene and the Eskimos and Aleuts, respectively. Lastly, individuals carrying mtDNA haplogroup B mtDNA moved out from the Asian coast to colonize all of the Pacific Islands (figure 1) [47,48].

This regionality of the mtDNA haplogroups is extraordinary in several ways. First, of all of the African diversity, only two mtDNA lineages (M and N) colonized the rest of the world. This striking fact was missed in the first paper to report an African origin of the mtDNA [49], since these authors included African-American mtDNAs in their ‘African’ sample and about 30 per cent of African-American mtDNAs are of European, Native American and Asian origin introduced after the arrival of Africans in the Americas [50]. Second, of all of the Asian mtDNAs, only three mtDNA lineages (A, C and D) moved to extreme northeast Siberia to found the Paleo-Indians. Third, and most surprising, the mtDNA sequence evolution rate is such that it produced important mtDNA evolutionary changes that coincide with the major human geographical transitions. It is hard to imagine that such associations occurred by chance. It is much more probable that mtDNA variation was intimately involved in facilitating the human adaptation to different regional environments.

Support for the hypothesis that the major human population transitions were facilitated by mtDNA variation comes from the observations that mtDNA lineages that appear together with human transitions often harbour important mtDNA functional changes [34,35,51]. For example, the founding mtDNA for macrohaplogroup N, which moved directly from sub-tropical Africa north into the European temperate zone, harboured two polypeptide variants: ND3 nt 10398 G>A (A114T) and ATP6 nt 8701 G>A (A59T)[47,48], which changed the mitochondrial membrane potential and Ca^2+^ metabolism [52]. By contrast, macrohaplogroup M, which remained in semi-tropical Southeast Asia, was not founded by distinctive polypeptide variants. Significant mitochondrial biochemical differences have also been reported between other haplogroups, for example, European haplogroups H and Uk [53,54].

That mtDNA variants might have been adaptive has been substantiated by studying mtDNA variation in Tibetans, who are adapted to high altitude and thus low oxygen tension. In Tibetans, the ratio of macrohaplogroup M to N mtDNAs is greater than in low-land Chinese. Furthermore, an mtDNA ND1 nt 3394C (Y30H) variant has been found to have arisen three independent times on different macrohaplogroup M mtDNAs in Tibetans, but never to have become established on any Tibetan macrohaplogroup N mtDNAs. The 3394C variant progressively increases in frequency in villages with increasing altitude. For example, haplogroup M9 mtDNA, which encompasses the 3394C variant, is present at less than 2 per cent of the mtDNAs at sea level but increases to almost 35 per cent of the mtDNAs in the highest Tibetan villages.

Biochemical analysis has shown that when the 3394C variant arises on macrohaplogroup N mtDNAs, specifically haplogroups B or F, it results in a 15–28% reduction in complex I-specific activity. However, when the 3394C allele is present on haplogroup M9 mtDNA, it is associated with a complex I activity that is equal to or greater than any of the macrohaplogroup N mtDNAs with the wild-type 3394T variant [40]. Hence, the 3394C variant is adaptive at high altitude when arising on macrohaplogroup M mtDNAs.

mtDNA haplogroups have also been correlated with predisposition to a wide range of metabolic and degenerative diseases, cancers and longevity [48,53,55,56]. For example, the ND1 nt 3394C (Y30H) variant, when arising on macrohaplogroup N mtDNAs in European populations, is associated with increased penetrance of the milder primary mtDNA mutations that cause Leber Hereditary Optic Neuropathy (LHON) [57,58]. Haplogroup F mtDNAs are associated with a predilection to diabetes [59] and analysis of the complex I-specific activities between mtDNA haplogroups B and F has revealed a 30 per cent lower complex I activity in haplogroup F [40]. Consequently, adaptive and clinically relevant mtDNA variants are also associated with functional changes in OXPHOS.

These data prove that at least some mtDNA variation is not neutral, but rather is functional and can be adaptive when present on the appropriate mtDNA background and environment. Yet the same variant in a different mtDNA background and/or environment can be deleterious and predispose to metabolic and/or degenerative diseases [34,35,51].

According to Kimura [12], the high mtDNA mutation rate should create many deleterious mutations resulting in a genetic load that could destroy the species. This concern is particularly apt for the mtDNA since every one of the genes of the mtDNA is critical for life. However, this concern is unfounded since the mammalian ovary has a pre-fertilization selection system that removes those proto-oocytes that harbour severe mtDNA mutations before they can be ovulated and fertilized [60,61]. Hence, these mutations never contribute to the genetic load of the species. The human female generates millions of proto-oocytes, but only successfully ovulates about 400. All the rest are eliminated by atresia. This apparent inefficiency might exist specifically to permit selection against severe mtDNA mutations, thus permitting a high mtDNA sequence evolution rate.

The high mtDNA mutation rate paired with the pre-fertilization selection means that de novo mtDNA mutations that subtly alter mitochondrial energy metabolism are constantly being introduced into the mammalian germline. These mtDNA variants then produce bioenergetic diversity that lets subpopulations within the species physiologically adapt to and occupy slightly different region energetic environments. Hence, mtDNA variation is an important component of the missing functional variation that allows subpopulations of humans and other animals to adapt to local environments.

Why does this system not work for nDNA anatomical gene mutations? The difference is that mitochondrial bioenergetics is manifest at the individual cellular level, so natural selection can act directly on the metabolism of the unfertilized oocyte and/or its associated nurse cells. By contrast, to express anatomical genes, the oocyte must be fertilized and progress through development to generate a fully formed individual before the mutation can be manifest and acted on by natural selection. Therefore, even the most deleterious nDNA anatomical mutations are passed into the population where they can contribute to the genetic load. The only way of minimizing the lethal effects of deleterious anatomical mutations is to keep the nDNA mutation rate very low. This means that nDNA mutations are generally too rare to be the initiating factors in intra-specific functional radiation.

What about mutations in nDNA-coded bioenergetic genes such as those detected by GWAS? As Kimura [12] predicted, most nuclear mutations result in lethal phenotypes, for example, those that cause Maturity Onset Diabetes of the Young (MODY) [31]. Less severe but still functionally significant metabolic gene mutations might be beneficial in peripheral energetic environments and become locally enriched by adaptive selection. However, if the energetic environment changes or the individuals migrate to a new environment these functional variants become maladaptive and are removed by purifying selection causing metabolic disease. Only the mildest phenotypic variants in bioenergetic loci escape selection and can become dispersed throughout populations and detected by GWAS [13,14].

## 5. Evolution and the interaction of Mendelian and mitochondrial genetics

Animals thus have two primary evolutionary strategies: an energetic strategy that changes rapidly and is optimal for intra-specific radiation into various regional energetic environments and an anatomical strategy that changes slowly and is optimal for inter-specific structural radiation for exploitation of new niches. The short-term energetic adaptive strategy is primarily driven by the high mtDNA mutation rate and the resulting rapid intra-specific energetic radiation. The long-term anatomical adaptive strategy is rooted in the low nDNA mutation rate, leading to subtle inter-specific anatomical alterations [7,32].

The continuously arising mtDNA functional variation allows subpopulations of animal species to occupy a range of energetic environments within the species' niche. Those subpopulations that occupy peripheral energetic environments for prolonged periods will amass multiple mtDNA variants permitting the adjustment to local energy resources and demands. As the mtDNA sequence evolves, the slower-evolving nDNA bioenergetic genes will follow to consolidate survival in the peripheral energetic environment and to maintain optimal nDNA and mtDNA interactions [62].

The nDNA variants can have a range of phenotypic consequences from large to small. Variants that are of physiological significance and thus phenotypic importance in moderately divergent environments include the specific genetic variants in PPARγ [22], PGC-1α [23,24] and UCP1-3 [16–19]. Examples of nDNA variants that permitted human adaptation to extreme environments are found among Tibetans, who have adapted to low oxygen tensions for tens of thousands of years. Adaptive Tibetan nDNA loci include an intronic SNP within endothelial Per-Arnt-Sim (PAS) domain protein 1 (EPAS1), also known as hypoxia-inducible factor 2α (HIF-2α). HIF-2α is a transcription factor that responds to hypoxia by inducing glycolysis and modifying mitochondrial energetics. The Tibetan SNP shows a 78 per cent frequency difference between Tibetan and Han samples [63]. Additional hypoxia pathway genetic variants found in Tibetans include the HIF-1α modifying prolyl hydroxylase domain-containing protein 2 (PHD2)(*EGLN1*) and the HIF-1α target gene *PPARA* (PPARα) [64].

However, when an environment changes those loci that were adaptive in the previous environment can become incompatible with the new environment. This can result in pathological conditions such as diabetes and obesity and these alleles are then removed by purifying selection. nDNA variants with more modest phenotypic effects can be retained and dispersed by stochastic processes.

Associations between mtDNA haplogroups and diabetes have been found when studying well-defined human populations [59]. However, studies attempting to correlate mtDNA SNPs to diabetes using an aggregate of multiple Europe and American populations have not been successful [65].

These considerations then provide a logic for linking the disparate observations about human genetic variation and the origin of intra-specific adaptive variation. The high mutation rate of the mtDNA together with the pre-fertilization ovarian selection provide a rapid adaptation system for each species to adjust to regional environmental changes. These variants have a very high functional effect and hence are relatively specific to particular regional environments and thus subpopulations. As a result, even relatively closely related populations have very different adaptive mtDNA variants and thus different mtDNA haplotypes, haplogroups and associated mtDNA SNPs. The high mtDNA mutation rate also means that the same adaptive variant can arise multiple independent times in disparate populations. However, in different genetic and environmental contexts the phenotypic effect of a particular mtDNA variant can be very different.

Once mtDNA variation has permitted a species' subpopulation to become established in a marginal energetic environment for prolonged periods, mutations that augment energetic adaptation also accumulate in the nDNA-coded bioenergetic genes. If the subpopulation remains indefinitely in the marginal environment, then sufficient nDNA changes will accumulate to render the mtDNA and nDNA variants of the isolated population incompatible with those of the parental population, causing speciation [62,66–73]. Consistent with this supposition, a number of nDNA OXPHOS gene variants have been found to correspond to primate speciation events [34,62,74–77].

As a subpopulation drifts away from the parent population energetically, new environmental energy reservoirs become accessible, frequently in significantly different physical forms. This selects for anatomical changes that optimize exploitation of the new energy reservoir.

Once the new species has arisen to exploit a new energy reservoir, the species will begin to expand its range to overlap that of the new energy resource. This will require the population to expand back into more median global energetic environments. The resulting reduction in the extreme environmental constraints will select for the return of the mitochondrial bioenergetic metabolism back to its more energy-efficient state. This revision back to the more energy-efficient mtDNA genotypes can occur relatively rapidly due to the high sequence evolution rate of the mtDNA and the strong selection for the most efficient OXPHOS system when calorie limitation is the primary selective factor.

Only a portion of the mtDNA polypeptide amino acids are amenable to adaptive mutations, since these mutations must alter the efficiency of the OXPHOS enzyme complex without altering the enzyme's structure or assembly. Since these amino acid substitutions do not affect the capacity of individuals within the species to successfully mate with each other, these mtDNA variants can be maintained within a species and are not the final arbiters of speciation. Thus, these adaptive mtDNA variants can be polymorphic within a species but be essentially invariant between species. The reversion of these adaptive polypeptide variants to a more universal mtDNA genotype following speciation may explain why the mtDNAs from one species can introgress into another very closely related species through a hybridization zone [78].

In addition to the adaptive mtDNA polypeptide variants, the high mtDNA mutation rate also generates mtDNA variants that alter the interaction between the mtDNA and nDNA subunits of the same OXPHOS complex. This class of mtDNA variants can also arise in marginal energy environments and may be beneficial in certain contexts. However, since they reduce the stability of the OXPHOS complexes, they produce a strong selective pressure for the appearance of nDNA mutations in the OXPHOS enzyme polypeptide that interacts with the mtDNA mutant polypeptide to permit optimal enzyme assembly and function. Once such pairs of complementary mtDNA and nDNA OXPHOS polypeptide variants become established in a peripheral environment, they will begin to limit the success of matings of individuals from the peripheral environment with those of the parental environment. Hence, these mtDNA variants can drive speciation via nuclear–cytoplasmic incompatibility [79].

In summary, there are two major classes of evolutionarily relevant mtDNA variants, one that permits expansion of the species range into energetically peripheral environments and another that leads to the genetic isolation of the peripheral population through the accumulation of complementary mtDNA and nDNA OXPHOS enzyme variants. As more of the nuclear–cytoplasmic subunit interaction variants accumulate, the genetic barrier between successful mattings of the parental and peripheral population increases until speciation becomes established. As the new species expands out from its peripheral energy environment, the adaptive mtDNA variants revert to permit the new species to optimally exploit its new energetic niche.

## 6. Energetic genetics and human health

The neutralist–selectionist debate arose because studies of nDNA genetic variation between human populations could not identify the required adaptive variation in the nDNA. The discovery and characterization of the mtDNA evolutionary system resolves this dilemma since it is mtDNA variation that provides the initial high impact variation for intra-specific adaption to different energy environments. The high mtDNA mutation rate permits physiological adaptation in the time span of thousands of years, which is the time frame for intra-specific radiation. Hence, it was mtDNA variation that permitted our ancestors to occupy new energetic environments, which is why mtDNA variation is regional and corresponds to major human geographical transitions.

Occupation of alternative energetic environments then enriched for rare nDNA variants, which consolidated the bioenergetic adaptation of the isolated animal populations to different energetic environments within the species' niche. Subsequent environmental changes and/or migration created gene–environment mismatches and bioenergetic stress that in humans manifests as metabolic disease. The most phenotypically significant adaptive loci generate the greatest functional disconnect and most severe disease when the environment changes. Hence, these are the risk factors for common diseases, not the much milder and ubiquitous variants detected across all populations by GWAS.

The clarification of the bioenergetic genetics of common metabolic diseases indicates that to identify high impact nDNA genetic loci for common diseases it will be necessary to sequence the entire genome of patients and controls from regional populations and look for novel regional genetic variants that correlate with the disease. With these new approaches, high impact loci should be found that will permit presymptomatic diagnosis in regional populations. Such advances will suggest new therapeutic approaches for the treatment and prevention of human disease [48].
